# Association between severity of different body regions and inadequate control of atopic dermatitis: results from a cross-sectional and longitudinal study

**DOI:** 10.3389/falgy.2026.1888094

**Published:** 2026-09-08

**Authors:** Zifan Li, Ruoxi Yu, Ying Lin

**Affiliations:** 1 The Second Clinical College, Guangzhou University of Chinese Medicine, Guangzhou, China; 2 Department of Dermatology, The Second Affiliated Hospital of Guangzhou University of Chinese Medicine (Guangdong Provincial Hospital of Chinese Medicine), Guangzhou, China; 3 Guangdong Clinical Research Center for Chinese Medicine Dermatology, Guangzhou, China; 4 Guangdong Provincial Key Laboratory of Chinese Medicine for Prevention and Treatment of Refractory Chronic Diseases, Guangzhou, China

**Keywords:** atopic dermatitis, Atopic Dermatitis Control Tool, body region, Eczema Area and Severity Index, real-world study

## Abstract

**Background:**

The Atopic Dermatitis Control Tool (ADCT) serves as a key metric for assessing atopic dermatitis (AD) management. However, the relationship between eczema severity in different body regions and inadequate control in AD patients has not been extensively researched. This study aimed to examine this association in both children and adults with AD and to further explore temporality and treatment effects through longitudinal follow-up.

**Methods:**

A cross-sectional questionnaire survey was conducted on pediatric and adult patients with AD in Guangzhou, China. The questionnaires were completed by the AD patients attending the Department of Dermatology at Guangdong Provincial Hospital of Chinese Medicine between March, 2024 and December, 2025. Additionally, a longitudinal sub-cohort of 96 AD patients with 24-week follow-up data was retrospectively analyzed.

**Results:**

A total of 713 AD patients completed the survey, including 175 children (aged 12–17 years old) and 538 adults. In the pediatric group, the severity of the head and neck and lower limbs was independently associated with inadequate disease control. In the adult group, the severity of the lower limbs was independently associated with poor disease control. In the longitudinal cohort, improvements in disease severity of the head and neck and lower limbs from baseline to week 24 were independently associated with ADCT-defined clinical improvement.

**Conclusion:**

The AD control status exhibits age-specific regional associations. Longitudinal data further demonstrate that reductions in disease severity in the head and neck and lower limbs were independently associated with clinical improvement. These findings are exploratory and require prospective validation before they can inform treatment prioritization.

## 1 Introduction

Atopic dermatitis (AD) is a persistent, relapsing inflammatory skin disorder. The core of its clinical management lies in achieving and maintaining long-term and stable disease control (1). Recently, clinical evaluation practices have undergone significant shifts, with an increasing emphasis on a comprehensive understanding of diseases’ effects on patients’ quality of life (QoL), moving beyond a sole focus on the severity of cutaneous manifestations (2). Patient-reported outcome measures (PROMs) have grown in significance (3). Among them, the Atopic Dermatitis Control Tool (ADCT) has become an important tool to judge the control status of AD due to its simplicity, efficiency, and rigorous validation (4). Its score can directly reflect the patient's real feelings in multiple dimensions such as itching, sleep, daily activities, and mood over the past week (5).

The clinical manifestations of AD show obvious age-related differences. In childhood, skin lesions are often distributed on the flexural areas such as antecubital and popliteal fossae, while in adults, lesions may be more localized or persistent in flexural areas but can also affect hands, neck, and face (6). The evolution of this distribution pattern suggests that there may be different pathophysiological mechanisms in different age stages (1). Of note, site-specific involvement may confer a disproportionate disease burden (7, 8). Ribero S et al. found that severity in the head and neck region was independently associated with worsening QoL in adults with AD, underscoring the importance of targeting specific bodily regions when evaluating clinical significance (9). At present, it is not clear what is the relationship between the severity of different body parts (head and neck, trunk, upper limbs, and lower limbs) and the comprehensive disease control. In addition, there are not enough studies including children and adults to systematically analyze the relationship between the severity of multiple parts and the disease control results measured by ADCT.

Therefore, this cross-sectional study examined age-specific patterns in the relationship between body region severity and inadequate control in pediatric and adult AD populations to inform clinical assessment approaches.

## 2 Materials and methods

### 2.1 Study design

This retrospective, real-world, and single-center study was conducted at Guangdong Provincial Hospital of Chinese Medicine from March 2024 to December 2025. The study comprised a cross-sectional analysis of AD patients and a retrospective longitudinal sub-cohort analysis of patients with uncontrolled AD at baseline. All procedures were performed in accordance with the ethical principles of the Declaration of Helsinki. The study was approved by the Institutional Review Board of Guangdong Provincial Hospital of Chinese Medicine (Approval No. BF2024-096).

### 2.2 Cross-sectional study population and assessments

Outpatients with a physician-confirmed diagnosis of AD who attended the Department of Dermatology during the study period were consecutively enrolled. The pediatric cohort included patients aged 12 to 17 years, as the ADCT has been validated for use in individuals aged 12 years and older (5). Demographic data, including age and sex, were recorded. Eligible patients were assessed with the use of the ADCT (10), the Eczema Area and Severity Index (EASI) (11) the Itch Visual Analogue Scale (VAS-Itch) (12), the Dermatology Life Quality Index (DLQI) (13), and the Patient-Oriented Eczema Measure (POEM) (14). Of note, in the pediatric cohort, the ADCT was self-reported by all participants. A score of ADCT ≥7 is indicative of the patient's condition being “uncontrolled” (10). Region-specific EASI scores were calculated for the head and neck, trunk, upper limbs, and lower limbs. Global EASI severity was categorized as follows: mild (EASI <7), moderate (EASI 7–21), and severe (EASI >21). All assessments were performed at a single visit.

Early onset of AD was defined as the onset of AD symptoms before 2 years of age. History of atopic diseases was defined as the presence of any one of the following conditions: urticaria, allergic rhinitis, asthma, or conjunctivitis. Family history of atopic diseases was defined as the presence of any of the above conditions (including AD) in first-degree relatives.

### 2.3 Longitudinal sub-cohort population, follow-up, and outcome definitions

A longitudinal sub-cohort was performed by retrospectively identifying patients from the same source population who had a baseline ADCT ≥7 and had available follow-up data at week 24. Patients in this sub-cohort received either traditional therapy (topical corticosteroids, calcineurin inhibitors, traditional Chinese medicine, or conventional systemic agents) or targeted systemic therapy (biologics or Janus kinase inhibitors) at the discretion of the treating physician. Follow-up assessments included repeated ADCT and region-specific EASI evaluations at baseline, week 12, and week 24. Of note, the “baseline” in this longitudinal analysis refers to the time of study enrollment (observational baseline), not the time of treatment initiation. Patients had already been receiving ongoing treatment for varying durations prior to their baseline assessment. Treatment selection was at the discretion of the treating physician based on clinical judgment, patient preference, and medication availability, resulting in non-randomized allocation. Therefore, this analysis is not designed to compare the effectiveness of different treatment strategies but rather to identify factors associated with clinical improvement in a real-world treatment context.

Clinical improvement at week 24 was defined as both (1) a ≥50% reduction in ADCT score from baseline (ADCT-50) and (2) an ADCT score <7 at week 24 (15), which represents a transition from uncontrolled to controlled status with a clinically meaningful individual change, according to the validation study by Pariser et al. (10). Changes in disease severity (*Δ*EASI) from baseline to week 24 were calculated separately for the head and neck, trunk, upper limbs, and lower limbs.

### 2.4 Statistical analysis

All statistical analyses were performed using R version 4.3.1. The normality of continuous variables was determined through the application of the Shapiro–Wilk test, with a *P* value >0.05 indicating a normal distribution. Normally distributed continuous variables were reported as mean ± standard deviation (SD), with independent sample t-tests utilized for comparisons. Non-normally distributed data were characterized by median [interquartile range (IQR)], while Mann–Whitney U tests served as the statistical method for comparative analysis. Categorical data were presented as *n* (%). To investigate potential associations among variables, Spearman's rank correlation coefficients were computed to assess the relationship between ADCT scores and global EASI, VAS-Itch, DLQI, and POEM measurements, with these analyses conducted separately for both pediatric and adult cohorts. In addition, Spearman's rank correlations were also performed between body region-specific EASI scores and between body region-specific *Δ*EASI scores in the longitudinal sub-cohort.

To identify independent associations, univariate logistic regression was first performed for all candidate variables. Variables with a univariate *P* value <0.1 were subsequently included in the multivariable logistic regression models, with inadequate control (ADCT ≥7) as the dependent variable. To distinguish region-specific associations from generalized disease severity, we performed a sensitivity analysis by adding global EASI severity as an additional covariate to the multivariable logistic regression models for both the pediatric and adult cohorts. Potential multicollinearity was assessed using Spearman's rank correlation coefficients between the variables and variance inflation factors (VIF). We defined the absence of severe multicollinearity as Spearman's rank correlation coefficients <0.7 and VIF values <5 for all included predictors. If the model exhibits severe multicollinearity, all variables from the univariate analysis will be subjected to Least Absolute Shrinkage and Selection Operator (LASSO) regression for variable selection using the glmnet package in R. All continuous predictors were standardized to a mean of zero and a standard deviation of one prior to fitting. The optimal penalty parameter (*λ*) was determined through 10-fold cross-validation, using binary deviation as the loss function. Considering the limited sample size, to prioritize the simplicity of the model, we selected *λ*.1se (the maximum *λ* value within one standard error range of the minimum deviation). LASSO was only used for variable selection. Variables with non-zero coefficients at *λ*.1se were subsequently included in the standard multivariate logistic regression model. Odds ratios (ORs) with 95% confidence intervals (CIs) were calculated for all logistic regression analyses. *P* < 0.05 was considered statistically significant for all analyses.

## 3 Results

**Figure 1:**
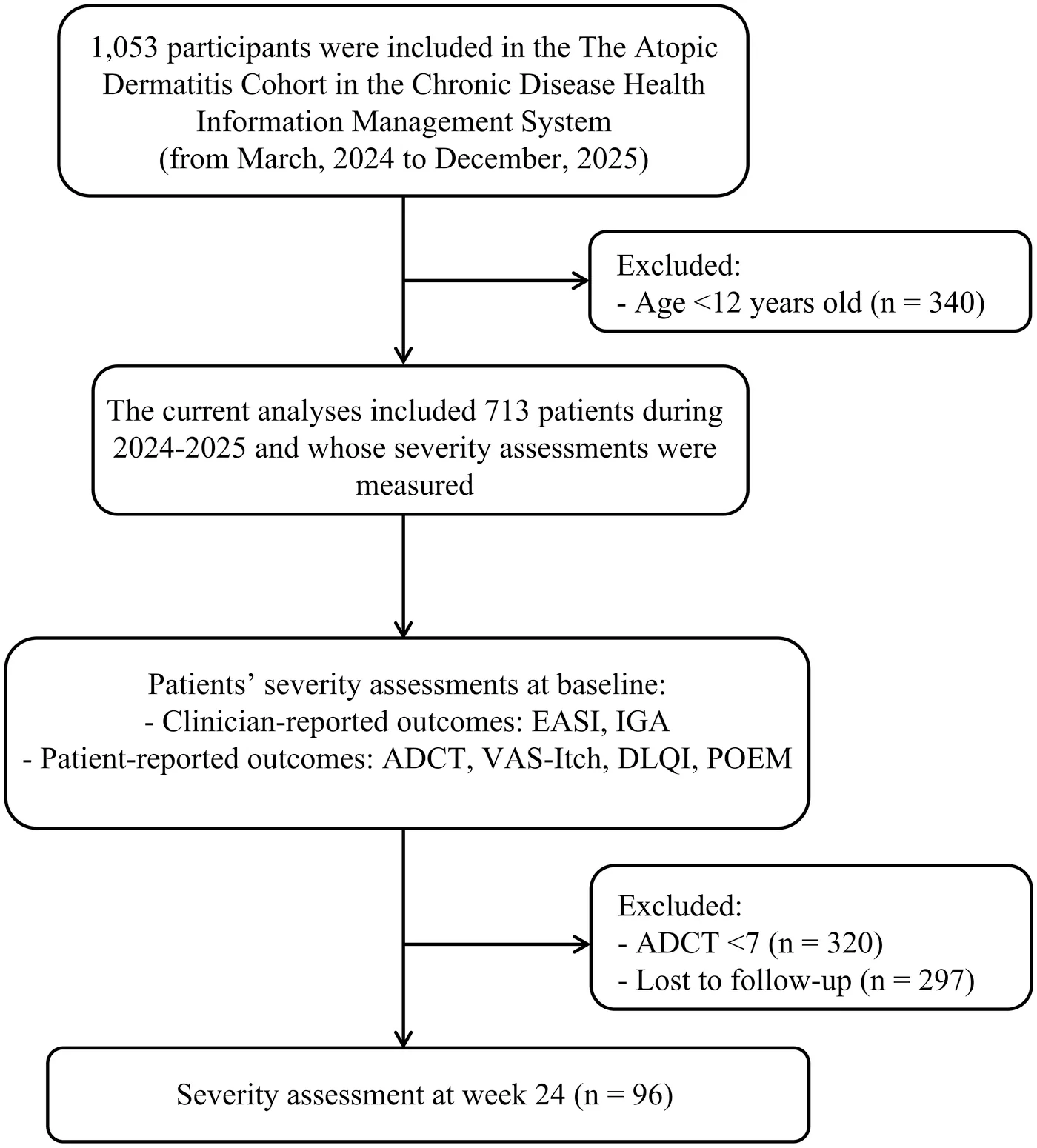
Flowchart of the participants selection.

The study comprised 713 AD patients (175 pediatric aged 12–17 years old, 538 adult), with a male predominance (55.4%). Median age was 22 years (IQR: 18–29), and median ADCT score was 7 (IQR: 4–12). PROMs included VAS-Itch (median 2, IQR: 2–4), DLQI (median 7, IQR: 3–11), and POEM (median 9, IQR: 4–15). Clinician-assessed EASI severity showed median score of 5.60 (IQR: 2.55–11.20) (Supplementary Table S1). Additionally, a longitudinal sub-cohort of 96 AD patients with baseline ADCT ≥7 and available 24-week follow-up data was retrospectively analyzed **(**Figure 1**)**.

### 3.1 Cross-sectional study

#### 3.1.1 Children group

The pediatric participants (*n* = 175) had a median age of 14 years (IQR: 12–16). Median age among pediatric participants (*n* = 175) was 14 years old (IQR: 12–16), with a female predominance (55.4%). Median ADCT score was 7 (IQR: 3–11). PROMs showed VAS-Itch of 2 (IQR: 2–4), DLQI of 6 (IQR: 3–9), and POEM of 8 (IQR: 3–14). EASI median was 5.10 (IQR: 2.55–8.98) (Supplementary Table S1).

**Figure 2:**
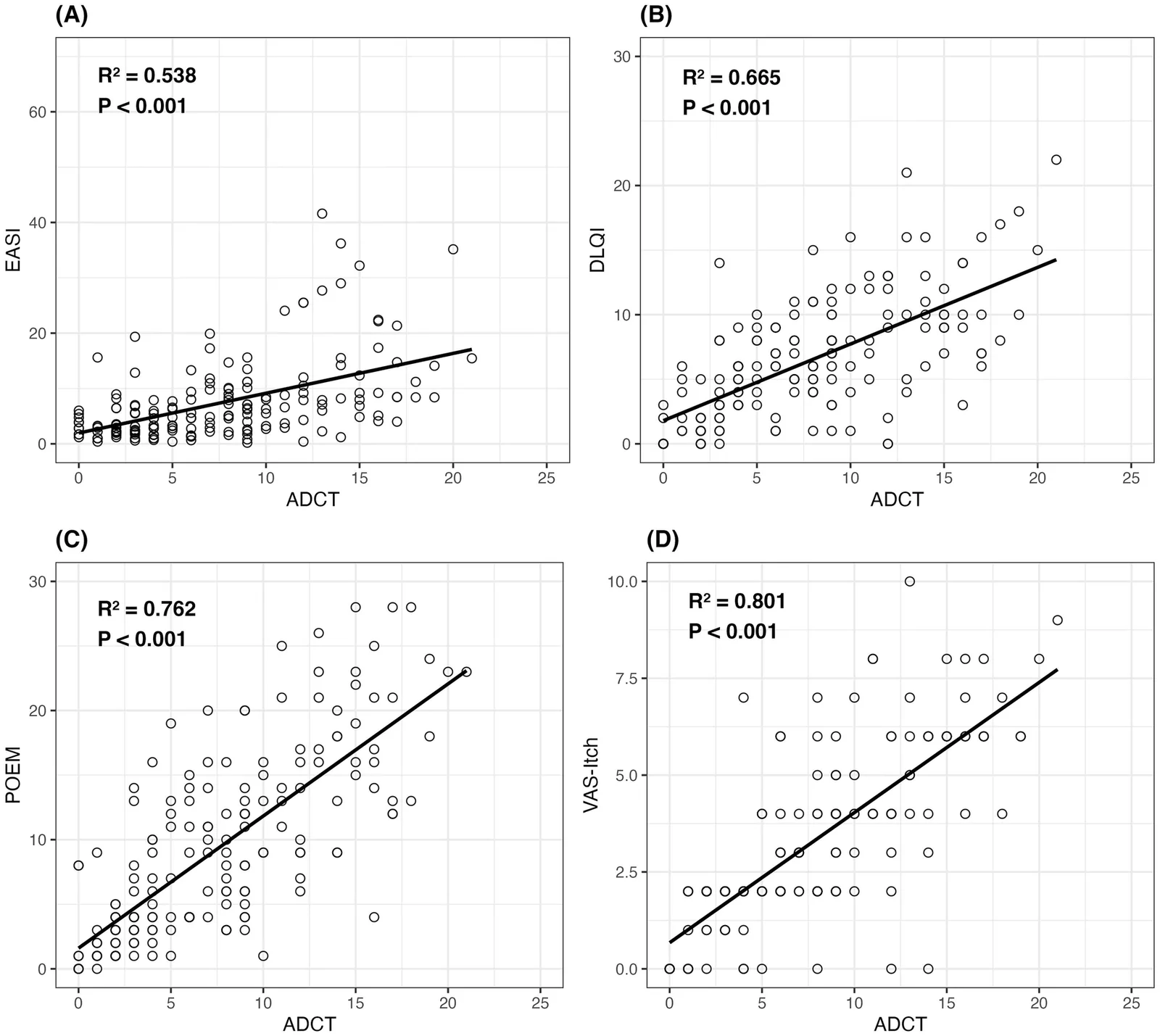
Correlation between ADCT and **(A)** EASI, **(B)** DLQI, **(C)** POEM, **(D)** VAS-itch in pediatric patients with AD.

Figure 2 reveals significant correlations between ADCT and all other assessed measures: EASI (r = 0.538, *P* = 0.001), DLQI (r = 0.665, *P* < 0.001), POEM (r = 0.762, *P* < 0.001), and VAS-Itch (r = 0.801, *P* < 0.001).

Pediatric AD patients were categorized as controlled (*n* = 84) or uncontrolled (*n* = 91) based on ADCT scores. The uncontrolled group demonstrated significantly older age [median 15 [IQR 12.5–17] vs. 14 [IQR 12–16] years, *P* = 0.028] and similar female proportion (61.5% vs. 48.8%, *P* = 0.123). All PROMs and clinician-assessed severity measures, including global and regional EASI scores, were significantly worse in the uncontrolled group (Table 1).

**Table 1:** Comparison of characteristics between patients with controlled and uncontrolled AD.

Characteristics	Children (*N* = 175)			Adult (*N* = 538)		
	Controlled AD (*N* = 84)	Uncontrolled AD (*N* = 91)	*P*	Controlled AD (*N* = 236)	Uncontrolled AD (*N* = 302)	*P*
Sex, n/N (%)						
Female	41/84 (48.8%)	56/91 (61.5%)	0.123	69/236 (29.2%)	152/302 (50.3%)	<0.001
Male	43/84 (51.2%)	35/91 (38.5%)		167/236 (70.8%)	150/302 (49.7%)	
Age	14 (12, 16)	15 (12.5, 17)	0.028	26 (21, 30)	25 (21, 30)	0.836
Early onset, n/N (%)	29/37 (78.4%)	26/41 (63.4%)	0.231	64/180 (35.6%)	72/176 (40.9%)	0.352
History of atopic diseases, n/N (%)	53/58 (91.4%)	41/53 (77.4%)	0.074	160/190 (84.2%)	164/206 (79.6%)	0.291
History of urticaria, n/N (%)	2/58 (3.45%)	7/53 (13.2%)	0.084	49/199 (24.6%)	50/211 (23.7%)	0.917
History of allergic rhinitis, n/N (%)	51/58 (87.9%)	34/53 (64.2%)	0.006	110/199 (55.3%)	112/211 (53.1%)	0.729
History of asthma, n/N (%)	0/58 (0.00%)	0/53 (0.00%)	/	33/190 (17.4%)	25/206 (12.1%)	0.184
History of conjunctivitis, n/N (%)	0/58 (0.00%)	4/53 (7.55%)	0.049	17/199 (8.54%)	29/211 (13.7%)	0.131
Family history of atopic diseases, n/N (%)	29/58 (50.0%)	42/53 (79.2%)	0.003	79/199 (39.7%)	122/211 (57.8%)	<0.001
VAS-Itch	2.0 (1.0, 2.0)	4.0 (3.0, 6.0)	<0.001	2.0 (1.0, 2.0)	4.0 (3.0, 6.0)	<0.001
DLQI	3.0 (1.0, 6.0)	8.0 (5.0, 11.0)	<0.001	3.5 (1.0, 6.0)	11.0 (7.5,15.0)	<0.001
POEM	4.0 (1.5, 7.0)	13.0 (9.0, 18.0)	<0.001	4.0 (2.0, 7.0)	14.0 (10.0, 20.0)	<0.001
Global EASI	3.0 (1.7, 5.5)	8.4 (4.9, 12.9)	<0.001	3.3 (1.6, 6.4)	9.0 (4.3, 15.8)	<0.001
Global EASI severity						
Mild	74 (88.1%)	35 (38.5%)	<0.001	181 (76.7%)	123 (40.7%)	<0.001
Moderate	10 (11.9%)	44 (48.4%)		48 (20.3%)	121 (40.1%)	
Severe	0 (0.00%)	12 (13.1%)		7 (3.00%)	58 (19.2%)	
Head & neck EASI	4.0 (1.0, 7.0)	6.0 (3.0, 15.0)	<0.001	4.0 (0, 12.0)	12.0 (2.0, 23.2)	<0.001
Trunk EASI	0 (0, 2.1)	6.0 (0, 12.0)	<0.001	3.0 (0, 8.0)	8.0 (2.0, 18.0)	<0.001
Upper limbs EASI	5.0 (3.0, 6.1)	8.0 (4.0, 15.5)	<0.001	3.0 (1.0, 5.5)	8.0 (3.0, 16.0)	<0.001
Lower limbs EASI	4.0 (1.9, 6.0)	8.0 (4.8, 15.0)	<0.001	3.0 (0, 5.2)	7.5 (3.5, 16.0)	<0.001

ADCT, Atopic Dermatitis Control Tool; VAS-Itch, visual analog scale for itch; DLQI, Dermatology Life Quality Index; POEM, Patient-Oriented Eczema Measure; EASI, Eczema Area and Severity Index.

*P* < 0.05 was considered significant.

Univariate logistic regression analysis revealed that advancing age (OR 1.20, 95% CI: 1.02–1.41; *P* = 0.024), family history of atopic diseases (OR 3.81, 95% CI: 1.68–9.13; *P* = 0.001), and increased severity in all four body regions (head & neck EASI OR 1.04, 95% CI: 1.03–1.06; trunk EASI OR 1.06, 95% CI: 1.04–1.08; upper limbs EASI OR 1.08, 95% CI: 1.06–1.11; lower limbs EASI OR 1.12, 95% CI: 1.09–1.16; all *P* < 0.001) were significantly associated with inadequate disease control in the pediatric cohort. A personal history of atopic diseases showed a protective association (OR 0.32, 95% CI: 0.09–0.94; *P* = 0.047) (Supplementary Table S2).

The multivariable logistic regression model, adjusted for age, history of atopic diseases, and family history of atopic diseases, revealed distinct associations between body region-specific severity and uncontrolled disease. Advancing age was independently associated with a higher likelihood of uncontrolled disease (OR 1.42, 95% CI: 1.07–1.96; *P* = 0.021). Family history of atopic diseases was also independently associated with a higher likelihood of uncontrolled disease (OR 10.00, 95% CI: 2.69–50.46; *P* = 0.001). History of atopic diseases showed no significant independent association (OR 0.46, 95% CI: 0.10–1.98; *P* = 0.297). Regarding specific body regions, increased severity in the head and neck (OR 1.14, 95% CI: 1.02–1.29; *P* = 0.024) and lower limbs (OR 1.16, 95% CI: 1.05–1.31; *P* = 0.006) remained independently associated with uncontrolled disease. Increased severity in the trunk (OR 1.10, 95% CI: 0.96–1.30; *P* = 0.195) and upper limbs (OR 0.95, 95% CI: 0.83–1.09; *P* = 0.485) did not show statistically significant associations (Table 2). To make these results easier to interpret clinically, we looked at the typical range of EASI scores in children whose disease was uncontrolled. For the head and neck, most uncontrolled children had scores between 3.0 and 15.0. A child whose head and neck EASI increased from the lower end to the upper end of this typical range would have roughly five times higher odds of having overall uncontrolled disease. Similarly, for the lower limbs (typical range 4.8 to 15.0), moving from the bottom to the top of that range was associated with about four to five times higher odds of being uncontrolled.

**Table 2:** Multivariable logistic regression analysis of the association between body region-specific EASI scores and inadequately controlled AD.

Characteristics	Total population (*N* = 713)			
	Children (*N* = 175)		Adult (*N* = 538)	
	OR (95% CI)	*P*	OR (95% CI)	*P*
Age	1.42 (1.07–1.96)^a^	0.021	/	/
Sex				
Male	/	/	1 [Reference]	<0.001
Female	/		3.85 (2.36–6.37)^b^	
History of atopic diseases	0.46 (0.10–1.98)^a^	0.297	/	/
Family history of atopic diseases	10.00 (2.69–50.46)^a^	0.001	1.79 (1.14–2.84)^b^	0.011
Head & neck EASI	1.14 (1.02–1.29)^a^	0.024	1.03 (0.99–1.06)^b^	0.072
Trunk EASI	1.10 (0.96–1.30)^a^	0.195	1.02 (0.98–1.06)^b^	0.249
Upper limbs EASI	0.95 (0.83–1.09)^a^	0.485	1.01 (0.97–1.05)^b^	0.390
Lower limbs EASI	1.16 (1.05–1.31)^a^	0.006	1.12 (1.07–1.17)^b^	<0.001

EASI, Eczema Area and Severity Index; OR, odds ratio; CI, confidence interval.

a adjusted for age, history of atopic diseases, family history of atopic diseases, head & neck EASI, trunk EASI, upper limbs EASI and lower limbs EASI.

b adjusted for sex, family history of atopic diseases, head & neck EASI, trunk EASI, upper limbs EASI and lower limbs EASI.

*P* < 0.05 was considered significant.

To further evaluate potential multicollinearity among the body region-specific EASI scores in the pediatric multivariable logistic regression model, Spearman's rank correlation coefficients between the four body regions were examined (Supplementary Figure S1A). The correlations were generally moderate to weak, with no evidence of strong collinearity. Additionally, variance inflation factors (VIF) for all included predictors were below 5 (Supplementary Figure S2), confirming that multicollinearity was not a significant concern in the model.

To assess whether the site-specific effects are independent of the overall severity of the disease, we stratified the global EASI by severity as a covariate and performed a sensitivity analysis. The results showed that the head and neck EASI remained independently associated with poor control (OR = 1.13, 95% CI: 1.01–1.28, *P* = 0.033), but the significance of the lower limbs EASI disappeared (OR = 1.12, 95% CI: 0.98–1.28, *P* = 0.082) (Supplementary Table S3).

#### 3.1.2 Adult group

Median age among adult participants (*n* = 538) was 26 years old (IQR: 21–30), with a male predominance (58.9%). Median ADCT score was 7 (IQR: 5–12). PROMs showed VAS-Itch of 2 (IQR: 2–4), DLQI of 7 (IQR: 3–11), and POEM of 9 (IQR: 4–15). EASI median was 5.88 (IQR: 2.55–12.15) (Supplementary Table S1).

**Figure 3:**
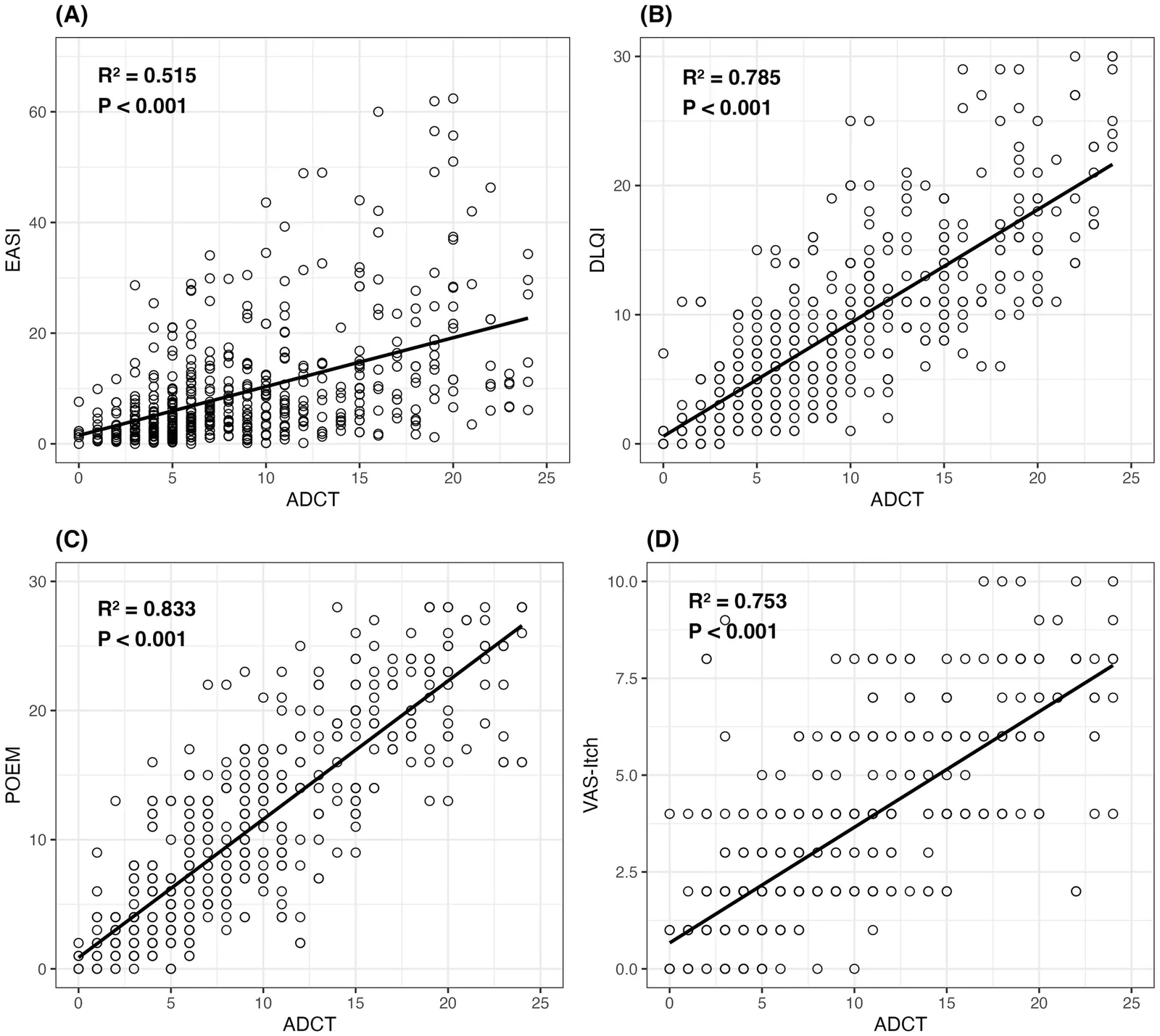
Correlation between ADCT and (A) EASI, (B) DLQI, (C) POEM, (D) VAS-Itch in adult patients with AD.

Figure 3 shows the correlations between ADCT and all other assessed measures: EASI (r = 0.515, *P* < 0.001), DLQI (r = 0.785, *P* < 0.001), POEM (r = 0.833, *P* < 0.001), and VAS-Itch (r = 0.753, *P* < 0.001).

Adult AD patients were categorized as controlled (*n* = 236) or uncontrolled (*n* = 302) based on ADCT scores. The uncontrolled group showed significantly higher female representation (50.3% vs. 29.2%, *P* < 0.001) but comparable median age (25 vs. 26 years old, *P* = 0.836). All PROMs and clinician-assessed severity measures, including global and regional EASI scores, were significantly worse in the uncontrolled group (Table 1).

Univariate logistic regression analysis revealed that female sex (OR 2.45, 95% CI: 1.71–3.52; *P* < 0.001), family history of atopic diseases (OR 2.08, 95% CI: 1.40–3.09; *P* < 0.001), and increased severity in all four body regions (head & neck EASI OR 1.04, 95% CI: 1.03–1.06; trunk EASI OR 1.06, 95% CI: 1.04–1.08; upper limbs EASI OR 1.08, 95% CI: 1.06–1.11; lower limbs EASI OR 1.12, 95% CI: 1.09–1.16; all *P* < 0.001) were significantly associated with inadequate disease control in the adult cohort. Age showed no significant association (OR 0.99, 95% CI: 0.97–1.01; *P* = 0.489) (Supplementary Table S2).

The multivariable logistic regression model, adjusted for sex and family history of atopic diseases, revealed distinct associations between body region-specific severity and uncontrolled disease. Female sex was independently associated with a substantially higher likelihood of uncontrolled disease compared to males (OR 3.85, 95% CI: 2.36–6.37; *P* < 0.001). Family history of atopic diseases was also independently associated with higher likelihood of uncontrolled disease (OR 1.79, 95% CI: 1.14–2.84; *P* = 0.011). Regarding specific body regions, increased severity in the lower limbs (OR 1.12, 95% CI: 1.07–1.17; *P* < 0.001) remained independently associated with uncontrolled disease. Among adults with uncontrolled AD, half had lower limbs EASI scores between 3.5 and 16.0. An increase from the lower to the upper end of this typical range corresponded to approximately four times higher odds of poor disease control. Increased severity in the head and neck (OR 1.03, 95% CI: 0.99–1.06; *P* = 0.072), trunk (OR 1.02, 95% CI: 0.98–1.06; *P* = 0.249) and upper limbs (OR 1.01, 95% CI: 0.97–1.05; *P* = 0.390) did not show statistically significant associations (Table 2).

To further evaluate potential multicollinearity among the body region-specific EASI scores in the adult multivariable logistic regression model, Spearman's rank correlation coefficients between the four body regions were examined (Supplementary Figure S1B). The correlations were generally moderate to weak, with no evidence of strong collinearity. Additionally, VIF for all included predictors were below 5 (Supplementary Figure S3), confirming that multicollinearity was not a significant concern in the model.

The results of sensitivity analysis showed that the lower limbs EASI was still significantly associated with poor control as defined by ADCT (OR = 1.13, 95% CI: 1.07–1.20, *P* < 0.001), confirming the independence of the adult lower limbs effect (Supplementary Table S3).

### 3.2 Longitudinal sub-cohort study

The flow of participant selection for the cross-sectional and longitudinal analyses is illustrated in Figure 1. 1,053 patients were screened from the Atopic Dermatitis Cohort in the Chronic Disease Health Information Management System. After excluding those aged <12 years old (*n* = 340), 713 patients (175 children and 538 adults) were included in the cross-sectional analysis. For the longitudinal sub-cohort, patients with a baseline ADCT score <7 (*n* = 320) and those lost to follow-up (*n* = 297) were further excluded, ultimately yielding 96 patients with available 24-week follow-up data.

To assess the comparability of the baseline ADCT across different subgroups, we stratified the baseline ADCT scores by age, sex, and treatment group **(**Supplementary Table S4**)**. Baseline ADCT scores were comparable across all subgroups (all *P* > 0.05), indicating that the ADCT ≥7 cutoff consistently identified patients with a similar level of perceived disease burden regardless of these demographic or treatment-related factors.

Baseline characteristics stratified by treatment group are presented in Supplementary Table S5. Patients receiving traditional therapy had numerically higher baseline global EASI (14.7 ± 13.2 vs. 10.1 ± 11.2, *P* = 0.090) and significantly higher head and neck EASI (1.67 ± 1.48 vs. 0.99 ± 1.16, *P* = 0.024) and trunk EASI (4.93 ± 5.27 vs. 2.72 ± 4.00, *P* = 0.038) compared to those receiving targeted systemic therapy. Conversely, baseline ADCT scores were nearly identical between the two groups (11.8 ± 6.16 vs. 11.8 ± 5.73, *P* = 0.998). No significant differences were observed in age, sex, or atopic history between the groups (Supplementary Table S5**)**. As the baseline in this cohort represents the time of study enrollment rather than treatment initiation, the observed differences in EASI scores likely reflect the cumulative therapeutic effects already achieved prior to enrollment, rather than differences in initial disease severity at the time of treatment prescription. Baseline characteristics and EASI changes in the longitudinal cohort are summarized in Table 3. Of these, 34 patients (35.4%) achieved clinical improvement at week 24, while 62 (64.6%) were classified as non-improved. It is noteworthy that the improved group in the longitudinal cohort had significantly higher baseline global EASI, reflecting a more severe disease burden at enrolment. This pattern is consistent with real-world clinical practice, where patients with greater disease activity are more often selected for targeted systemic therapy. The improved group had significantly higher baseline global EASI, lower limbs EASI. A higher proportion of patients in the improved group received targeted systemic therapy, particularly with more frequent current use of dupilumab. No significant differences were observed in sex, age, early onset, or history of atopic diseases between the two groups. In addition, the improved group demonstrated significantly greater reductions in all four body regions from baseline to week 24 (Table 3).

**Table 3:** Baseline characteristics and EASI changes in the longitudinal cohort (*N* = 96).

Characteristics	Total population (*N* = 96)		*P*
	Improved (*N* = 34)	Non-improved (*N* = 62)	
Sex, n/N (%)			
Female	11/34 (32.4%)	31/62 (50.0%)	0.147
Male	23/34 (67.6%)	31/62 (50.0%)	
Age	24.7 ± 8.73	23.4 ± 9.82	0.490
Early onset, n/N (%)	11/34 (32.4%)	24/62 (38.7%)	0.691
History of atopic diseases, n/N (%)	28/34 (82.4%)	48/62 (77.4%)	0.759
History of urticaria, n/N (%)	5/34 (14.7%)	15/62 (24.2%)	0.405
History of allergic rhinitis, n/N (%)	24/34 (70.6%)	39/62 (62.9%)	0.594
History of asthma, n/N (%)	5/34 (14.7%)	4/62 (6.45%)	0.272
History of conjunctivitis, n/N (%)	2/34 (5.88%)	3/62 (4.84%)	1.000
Family history of atopic diseases, n/N (%)	21/34 (61.8%)	39/62 (62.9%)	1.000
Treatment, n/N (%)			
Traditional therapy	8/34 (23.5%)	26/62 (41.9%)	0.114
Targeted systemic therapy	26/34 (76.5%)	36/62 (58.1%)	
Current use of immunosuppressant, n/N (%)	0/34 (0.00%)	3/62 (4.84%)	0.550
Current use of Dupilumab, n/N (%)	23/34 (67.6%)	28/62 (45.2%)	0.058
Current use of JAK inhibitor, n/N (%)	3/34 (8.82%)	8/62 (12.9%)	0.741
Global EASI at baseline	15.5 ± 14.8	9.64 ± 9.81	0.044
Head & neck EASI at baseline	1.48 ± 1.57	1.10 ± 1.15	0.224
Trunk EASI at baseline	4.52 ± 5.54	2.95 ± 3.91	0.054
Upper limbs EASI at baseline	3.10 ± 3.06	1.93 ± 2.15	0.148
Lower limbs EASI at baseline	6.41 ± 6.50	3.66 ± 4.28	0.032
ADCT at baseline	13.1 ± 5.69	11.0 ± 5.86	0.092
*Δ* Head & neck EASI	1.12 ± 1.33	0.07 ± 1.28	<0.001
*Δ* Trunk EASI	3.21 ± 3.99	0.04 ± 3.81	<0.001
*Δ* Upper limbs EASI	2.11 ± 2.39	−0.14 ± 2.33	<0.001
*Δ* Lower limbs EASI	4.71 ± 5.84	0.13 ± 3.97	<0.001

*Δ* = change from baseline to Week 24. JAK, Janus kinase; EASI, Eczema Area and Severity Index; ADCT, Atopic Dermatitis Control Tool.

*P* < 0.05 was considered significant.

Univariate logistic regression analysis revealed that higher baseline global EASI (OR 1.04, 95% CI: 1.01–1.08; *P* = 0.029), and increased baseline severity in the upper limbs (OR 1.19, 95% CI: 1.01–1.43; *P* = 0.040), and lower limbs (OR 1.10, 95% CI: 1.01–1.20; *P* = 0.021) were significantly associated with clinical improvement at week 24. In addition, greater reductions in disease severity from baseline to week 24 in the head and neck (OR 1.87, 95% CI: 1.32–2.82), trunk (OR 1.23, 95% CI: 1.09–1.41), upper limbs (OR 1.59, 95% CI: 1.26–2.13), and lower limbs (OR 1.23, 95% CI: 1.11–1.39; all *P* ≤ 0.001) were strongly associated with clinical improvement. Targeted systemic therapy showed a borderline significant association (OR 2.34, 95% CI: 0.94–6.29; *P* = 0.075) **(**Supplementary Table S6**)**.

**Figure 4:**
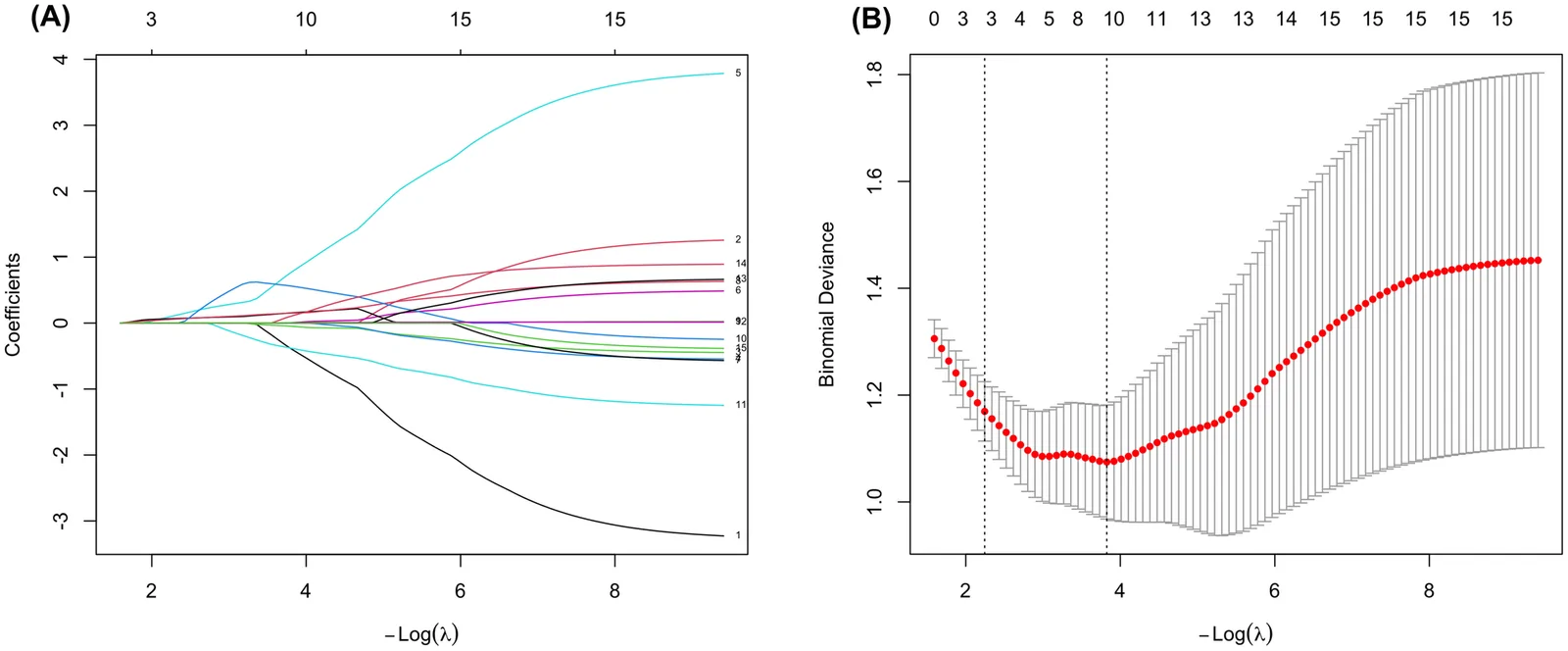
The results of variable selection via LASSO regression. **(A)** Cross-validation plot for the penalty term. **(B)** LASSO coefficient profiles of the 15 variables.

To construct the multivariable logistic regression model for clinical improvement at week 24, variables with a univariate *P* < 0.1 were initially included. Before constructing the multivariable model, we examined the correlations among the four body region-specific*Δ*EASI scores **(**Supplementary Figure S4**)**. Moderate correlations were observed. These correlations, while not extremely high, were sufficiently strong to cause severe multicollinearity in the initial multivariable logistic regression model (VIF >5, as shown in Supplementary Figure S5). To address this issue, all variables from the univariate analysis were subjected to LASSO regression for variable selection, with the penalty parameter (*λ*) determined by 10-fold cross-validation **(**Figure 4**)**. The effective variables retained by LASSO were entered into the final multivariable logistic regression model. The results demonstrated that reductions in head and neck EASI (OR 1.60, 95% CI: 1.03–2.63; *P* = 0.016) and lower limbs EASI (OR 1.18, 95% CI: 1.09–1.54; *P* = 0.043) from baseline to week 24 were significantly associated with ADCT-defined clinical improvement at 24 weeks. In patients who improved, the reduction in head and neck EASI typically ranged from about 0.0 to 1.8 points; achieving the full typical reduction was associated with roughly 2.5-fold higher odds of achieving good overall disease control. For the lower limbs, the typical reduction ranged from about 1.2 to 8.9 points, corresponding to approximately 5-fold higher odds of success. However, reductions in upper limbs EASI were not significantly associated with clinical improvement (OR 1.15, 95% CI: 0.85–1.62; *P* = 0.372) (Table 4). Additionally, all included predictors had VIF <5 (Supplementary Figure S6), confirming that multicollinearity was not a significant concern in the model.

**Table 4:** Multivariable logistic regression analysis of the association between body region-specific EASI score changes and clinical improvement in AD at week 24 (following LASSO variable selection).

Characteristics	Total population (*N* = 96)	
	OR (95% CI)	*P*
*Δ* Head & neck EASI	1.60 (1.03–2.63)	0.016
*Δ* Upper limbs EASI	1.15 (0.85–1.62)	0.372
*Δ* Lower limbs EASI	1.18 (1.09–1.54)	0.043

*Δ*= change from baseline to Week 24.

EASI, Eczema Area and Severity Index; OR, odds ratio; CI, confidence interval.

*P* < 0.05 was considered significant.

## 4 Discussion

AD is a chronic inflammatory disorder with systemic impact beyond skin manifestations, significantly compromising quality of life and frequently associated with psychological comorbidities and additional inflammatory conditions (1). In recent years, moderate to severe AD can be effectively managed by various treatments, so as to achieve long-term control and prevent recurrence (16, 17). Consequently, tools for evaluating long-term AD management are increasingly necessary. Effectively managing AD requires evaluating multiple dimensions. In order to meet this demand, ADCT is designed so that patients can evaluate their overall disease control by themselves. The format of ADCT is simple, clear and easy to understand, and it is also convenient for daily medical treatment. Its simplicity makes it particularly easy to use in medical consultations to facilitate shared decision making, including collaborative setting of treatment goals (10). Based on these advantages, ADCT has been proved to be a tool suitable for adults and adolescents aged 12 and above with AD (5, 18). We preferentially selected ADCT over clinician-assessed instruments such as SCORing Atopic Dermatitis (SCORAD) because the primary focus of this study was patient-perceived disease control. ADCT is a validated patient-reported outcome measure that captures multiple dimensions of control from the patient's perspective, whereas SCORAD is a composite clinician-assessed severity score. Given the growing emphasis on PROMs in AD management and shared decision-making, ADCT aligns more closely with our research question of identifying factors associated with inadequate control as experienced by patients.

At present, the ADCT has been verified in many countries and has excellent measurement performance. The dependability, authenticity, and adaptability of ADCT were substantiated in a longitudinal investigation. Additionally, the study determined that a significant cutoff for individual internal change is 5 points (10). In another study, Pariser et al. identified an ADCT score≥7 as the optimal threshold for identifying patients with uncontrolled AD (10). Furthermore, a Chinese study has demonstrated that the Chinese adaptation of the ADCT exhibits solid reliability, validity, and responsiveness, making it suitable for managing the condition of Chinese patients with AD. Several studies have found that ADCT is significantly correlated with DLQI, POEM, and EASI (19–21). This study corroborates prior research findings. Although there is an obvious connection between EASI and ADCT, this connection is not as strong as that between PROMs and ADCT. The moderate correlations observed between ADCT and global EASI (r = 0.538 in children and r = 0.515 in adults) contrasted with the stronger correlations observed with PROMs, including POEM (r = 0.762–0.833) and DLQI (r = 0.665–0.785). This pattern indicates that while there is some shared variance, the ADCT primarily captures unique subjective dimensions of disease control, including effects on sleep, daily activities, mood, and patients’ overall perception of their condition. These aspects are not fully reflected by the clinician-assessed EASI, which focuses more on objective morphological features. Thus, the relationship between regional severity and ADCT-defined inadequate control likely reflects complementary patient-centered mechanisms rather than simple construct redundancy.

In the cross-sectional analysis, the severity of the head and neck and lower limbs was independently associated with inadequate disease control in pediatric patients. In adults, lower limbs severity showed the strongest independent association, while head and neck severity exhibited a borderline association. In children, head and neck severity and lower limbs severity remained independently associated. This discovery is not only consistent with the previous research, but also further expands it. Previous studies have found that the severity of head and neck is the key factor affecting the decline of QoL (22). Based on this, our research further shows that its influence is not limited to the DLQI, but also has a significant correlation with the lack of disease control. Studies have shown that AD in the head and neck usually does not respond so well to biological agents like dupilumab compared with other parts (23). Because of its special location, it is often difficult to treat AD in the head and neck, so it may often be more uncontrolled (22). In the two age groups, the severity of symptoms of lower limbs is closely related to the obstinacy of the disease, which shows that lower limbs are the key parts of the disease that are persistent and difficult to control, and have a great influence on the overall disease control. Specific body regions matter in AD because lesions on visible or high-impact sites (such as the head and neck) or functionally important areas (such as the lower limbs) can disproportionately impair QoL, sleep, self-esteem, and daily activities, even when global severity scores appear moderate. Inadequate control in these regions is associated with substantial residual disease burden and may contribute to treatment dissatisfaction. Supporting this, the JADE COMPARE study demonstrated rapid and persistent improvement in signs and symptoms of moderate-to-severe AD across body regions with abrocitinib, highlighting the clinical relevance of region-specific responses to therapy (26). Likewise, Wei et al. documented the extensive consequences of inadequate disease control among adults with a history of moderate-to-severe AD, including poorer QoL, greater work impairment, and higher itch and sleep disturbance (27). A recent review further emphasized unmet needs related to clinical variability and regional involvement in AD management (28). Then the sensitivity findings suggest that, in children, the contribution of lower limb severity may be partly collinear with or mediated by global disease severity, while head and neck involvement appears to exert an effect that is more independent of overall severity. In adults, lower limbs severity retains a robust, independent relationship with patient-perceived control even after accounting for global EASI severity, reinforcing its particular clinical importance in this age group.

In addition to body region-specific severity, several demographic and atopic background factors demonstrated independent associations with inadequate disease control. In the pediatric cohort, advancing age was independently associated with a higher likelihood of uncontrolled disease. A previous study pointed out that age is the key factor affecting AD burden in children, and the increase of age shows a strong correlation with the higher QoL scores reported by patients and caregivers (24). Family history of atopic diseases emerged as a strong independent risk factor, particularly pronounced in children, and to a lesser extent in adults, underscoring the role of genetic susceptibility and shared familial environment. Interestingly, in univariate analysis, a personal history of other atopic diseases showed a protective association with uncontrolled AD in children, a counterintuitive finding. This apparent protective effect may be explained by detection or surveillance bias. Children with known atopic comorbidities are likely to be under closer dermatological follow-up and may have more proactive treatment regimens, leading to better control of AD itself. Alternatively, it could reflect a form of collider bias or selection bias inherent to hospital-based cross-sectional studies, where the sickest children without other atopic diseases are overrepresented. Nevertheless, this association lost statistical significance after adjusting for disease severity and family history in the multivariable model, suggesting that the univariate finding was confounded and should not be overinterpreted. In addition, we also showed that females had significantly higher ADCT scores and were more likely to perceive their AD as uncontrolled compared to males in the two groups. This is consistent with a previous study (25). The strong association of female with uncontrolled AD indicates that women may bear a greater burden of disease, which may be related to hormonal factors, psychosocial stress, medical treatment behavior or differences in symptom reports. Interestingly, the observed age and sex patterns across the two cohorts raise the possibility of hormonal influences on disease control. In the pediatric group, controlled children were slightly older than uncontrolled children, a difference that coincided with the peripubertal and adolescent age range. It is plausible that the progressive increase in androgen production during puberty may contribute to improved disease control in some adolescents, as androgens are known to exert immunomodulatory effects and enhance skin barrier function. In the adult cohort, a higher proportion of males achieved controlled status compared to females. This sex discrepancy, together with the predominance of head and neck involvement in uncontrolled adults, a region particularly sensitive to hormonal influences, further supports a potential role of sex hormones in AD control. These hypotheses are speculative and warrant dedicated prospective studies. A related clinical observation is that the ankle area is often the slowest to improve with treatment. Whether this reflects relative androgen insensitivity in the distal lower extremities, or other local factors such as friction, occlusion, or impaired barrier recovery, cannot be determined from our data, but this hypothesis merits further investigation. In contrast, a personal history of other atopic diseases and early onset of AD showed associations in univariate analyses but lost independent significance in multivariable models, indicating that their effects may be largely mediated through disease severity, familial predisposition, or other confounders.

We conducted a detailed retrospective longitudinal analysis of a real-world sub-cohort of 96 patients who had uncontrolled AD at baseline and complete 24-week follow-up data to further explore temporality and treatment effects. After addressing multicollinearity through LASSO variable selection, improvements in disease severity of the head and neck and lower limbs from baseline to week 24 were independently associated with ADCT-defined clinical improvement. These temporal findings suggest that reductions in disease activity in the head and neck and lower limbs may play a critical role in achieving overall disease control.

The results of this study show that when ADCT is used to evaluate disease control in clinical environment, it should be evaluated according to age, and special attention should be paid to “high-impact” body parts. For children with critical or poor ADCT scores, it is necessary to carefully evaluate the head, neck and lower limbs, because the skin lesions in these parts may be the key factor leading to the disease out of control. Similarly, for adult patients, priority should be given to evaluating the severity of the lower limbs. This approach combines patient's subjective report with objective clinical evaluation, and moves towards a more comprehensive and personalized evaluation model. Longitudinal data further indicate that reductions in disease severity in the head and neck and lower limbs were independently associated with clinical improvement evaluated by ADCT. These observational associations are hypothesis-generating rather than confirmatory.

This study has several advantages. It includes a large sample size of 713 patients in the cross-sectional analysis supplemented by a longitudinal sub-cohort of 96 patients, providing sufficient statistical power. To our knowledge, this is the first study to systematically evaluate the associations between body region-specific disease severity and ADCT-defined disease control in both pediatric and adult populations simultaneously. Multivariable adjustments for key confounders were performed to minimize bias. The incorporation of real-world longitudinal data with detailed treatment information and serial regional severity assessments overcomes key limitations of the cross-sectional design by providing temporal evidence and directly addressing treatment-related confounding, thereby strengthening causal inference. Despite these strengths, several limitations should be acknowledged. First, the single-center design at a tertiary hospital in China may limit the generalizability. Second, although the longitudinal sub-cohort offers important temporal insights, its retrospective nature may introduce selection bias. Third, the longitudinal analysis was limited by a modest sample size (*n* = 96, with only 34 patients achieving clinical improvement), which led to model overfitting concerns and wide confidence intervals. Thus, these longitudinal findings should be regarded as exploratory. Fourth, missing data for several variables (e.g., early onset, history of atopic diseases) may have introduced bias. Fifth, despite our efforts to adjust for multiple confounders (including age, sex, atopic history, baseline disease severity, and treatment type), we did not systematically collect data on the quantity, frequency, or type of emollients and syndets used by patients. Given that adherence to basic skin care can influence disease control, the lack of this information may introduce residual confounding, particularly in the longitudinal analysis where treatment regimens were not standardized. In particular, the longitudinal analysis is susceptible to confounding by indication, as the choice of targeted systemic therapy versus traditional therapy was determined by the treating physician based on disease severity and other unmeasured factors (e.g., patient preference, prior treatment failure, or comorbid conditions). Therefore, the observed associations with treatment should not be interpreted as causal comparative effectiveness. Sixth, we did not perform formal adjustment for multiple comparisons (e.g., Bonferroni correction) given the hypothesis-generating nature of this study and the risk of inflating type II error. Consequently, findings with borderline significance should be interpreted cautiously and require independent validation. Future multicenter, prospective longitudinal studies are warranted to validate these findings and further elucidate the role of region-specific severity in guiding personalized AD management.

## 5 Conclusion

In conclusion, for pediatric AD patients, the severity of the disease in the head, neck and lower limbs was independently associated with inadequate disease control. For adults patients, the severity of lower limbs was also independently associated with inadequate control. Longitudinal evidence further demonstrates that reductions in disease severity in head and neck and lower limbs regions were independently associated with clinical improvement, but these results are exploratory due to limited sample size and should be interpreted cautiously. Prospective validation is needed before these results can inform treatment prioritization or clinical decision-making.

## Data Availability

The raw data supporting the conclusions of this article will be made available by the authors, without undue reservation.
