# Supplementary material for: Association between severity of different body regions and inadequate control of atopic dermatitis: results from a cross-sectional and longitudinal study
Source: Front Allergy. 2026 Sep 8;7:1888094. doi: 10.3389/falgy.2026.1888094 (PMC13551416; doi:10.3389/falgy.2026.1888094)
Supplement: Supplementary file 1 [file Datasheet1.pdf]

Supplementary Material

1 Supplementary Figures and Tables

1.1 Supplementary Figures

Supplementary Figure 1. Spearman's rank correlation coefficients between body region–specific EASI scores for (A) pediatric patients with AD and (B) with adult patients with AD.

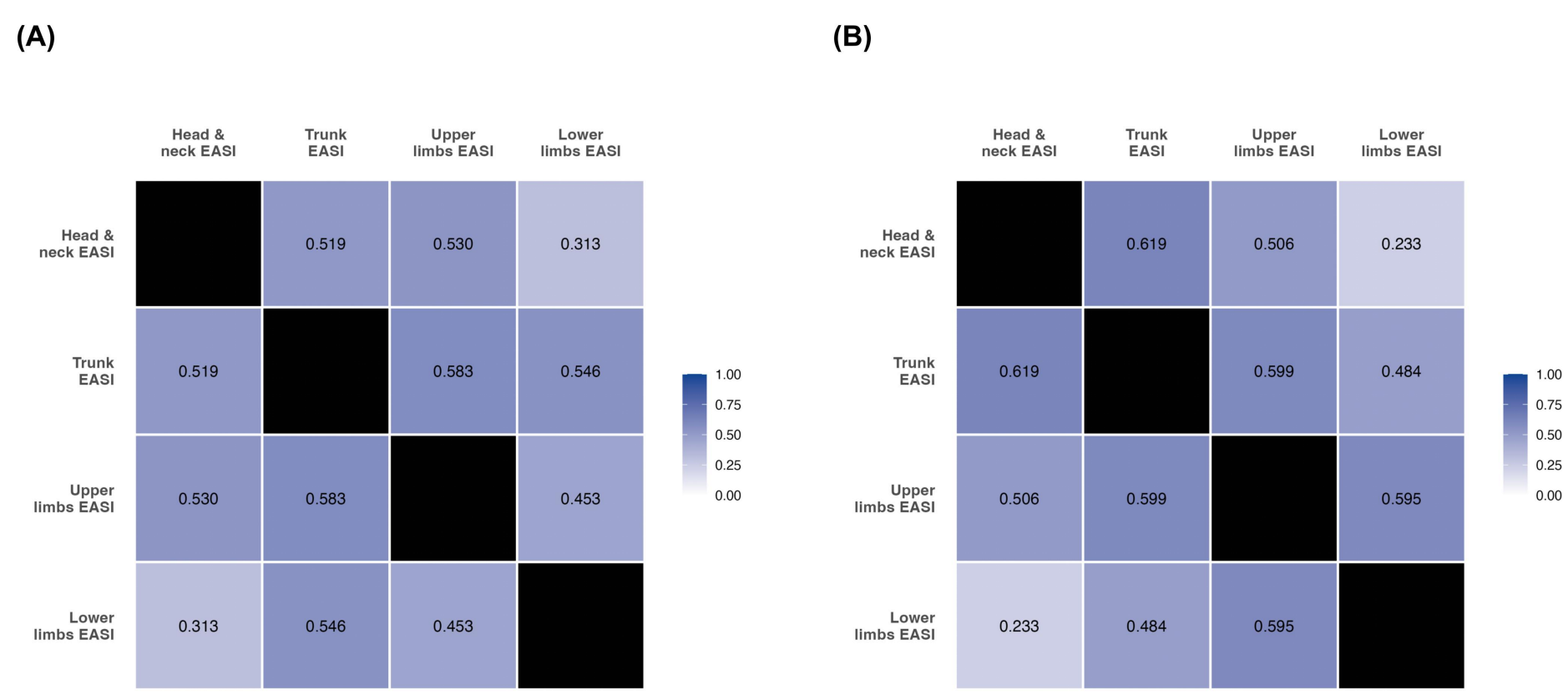

Supplementary Figure 2. Variance Inflation Factors (VIF) for predictors in multivariable logistic regression of body region-specific EASI scores with inadequately controlled AD in pediatric patients.

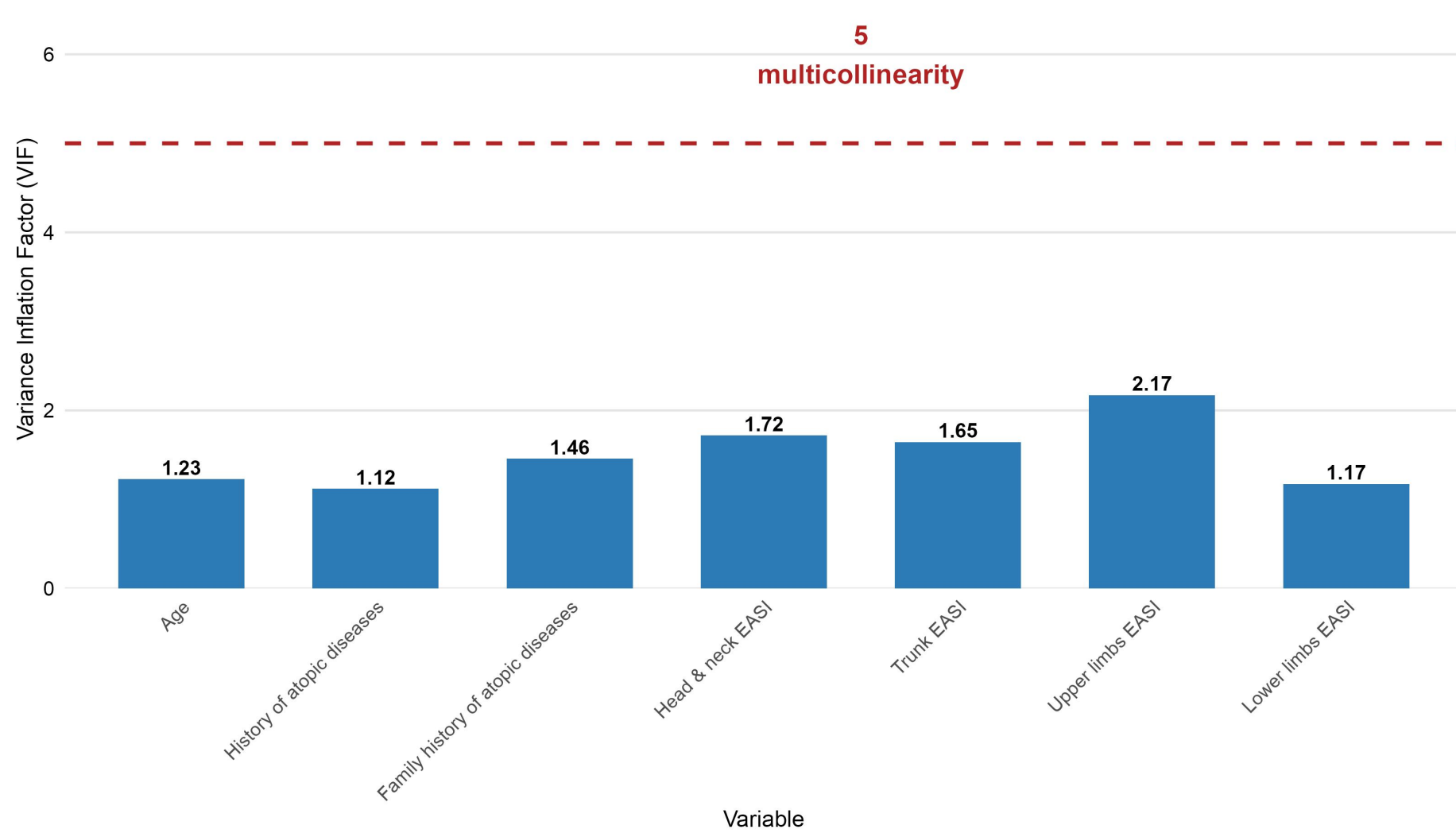

Supplementary Figure 3. Variance Inflation Factors (VIF) for predictors in multivariable logistic regression of body region-specific EASI scores with inadequately controlled AD in adult patients.

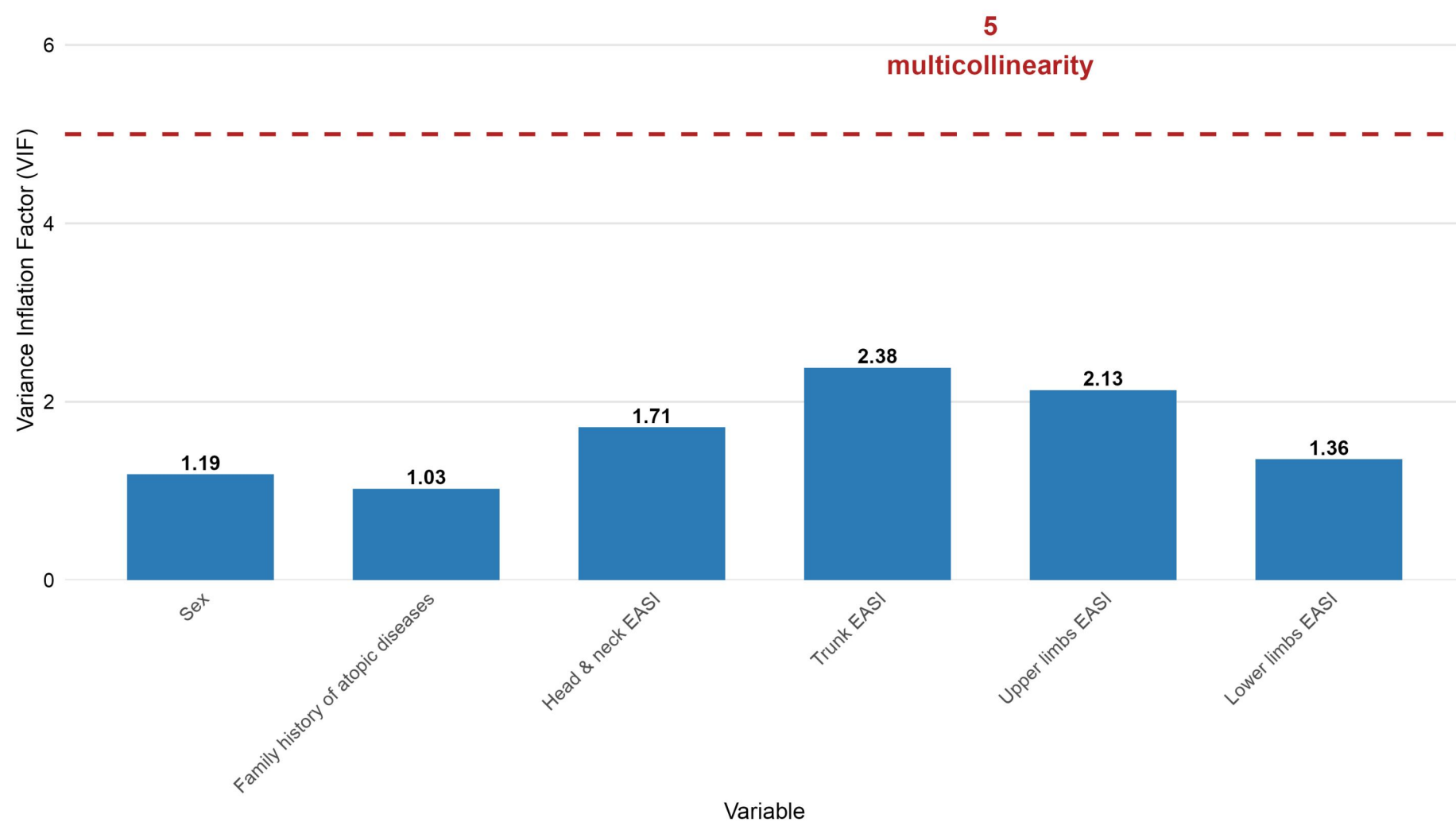

Supplementary Figure 4. Spearman's rank correlation coefficients between body region–specific EASI scores changes for AD patients in the longitudinal cohort.

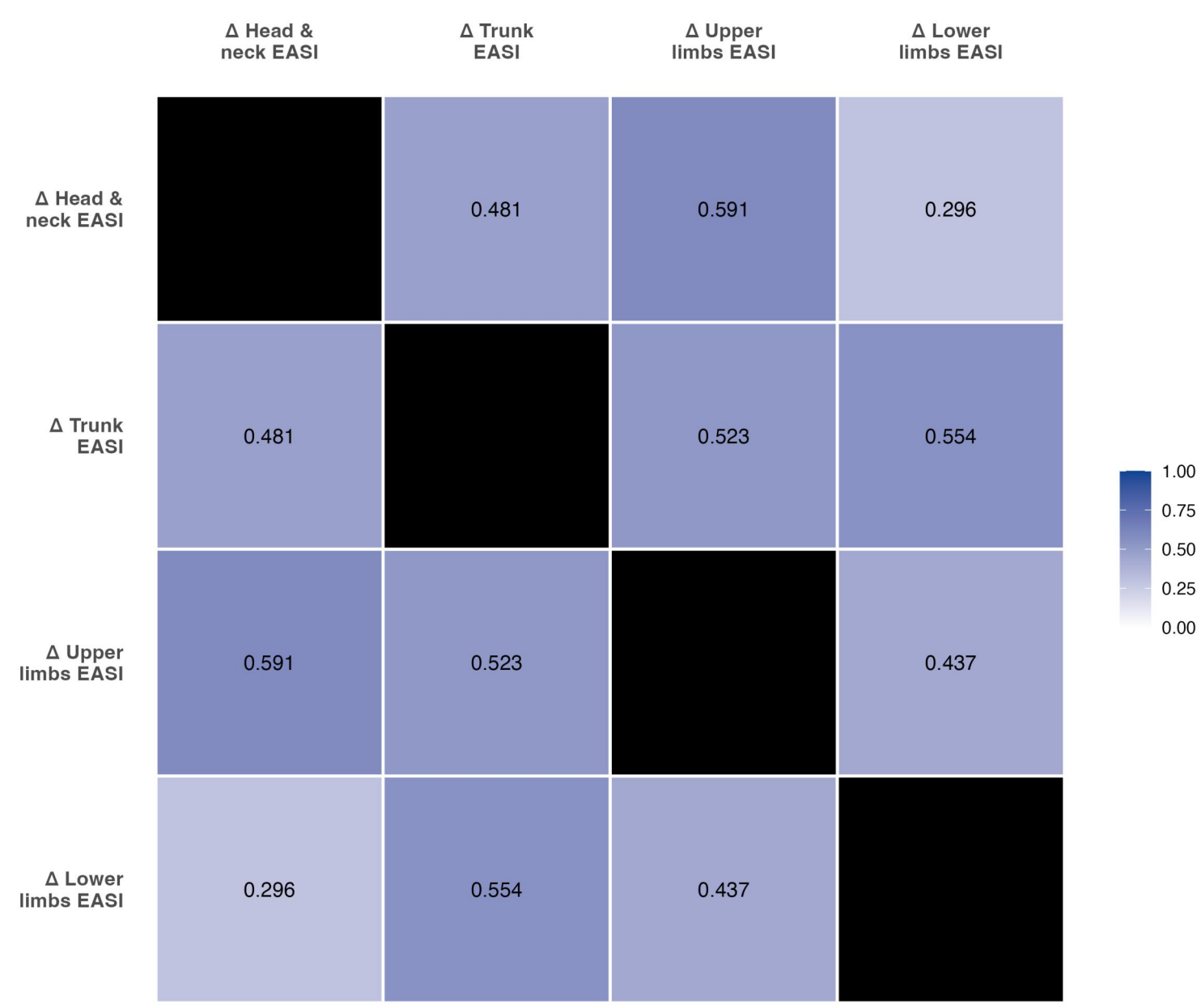

Supplementary Figure 5. VIF for predictors in multivariable logistic regression assessing body region-specific EASI score changes and clinical improvement in AD at Week 24, before applying LASSO variable selection.

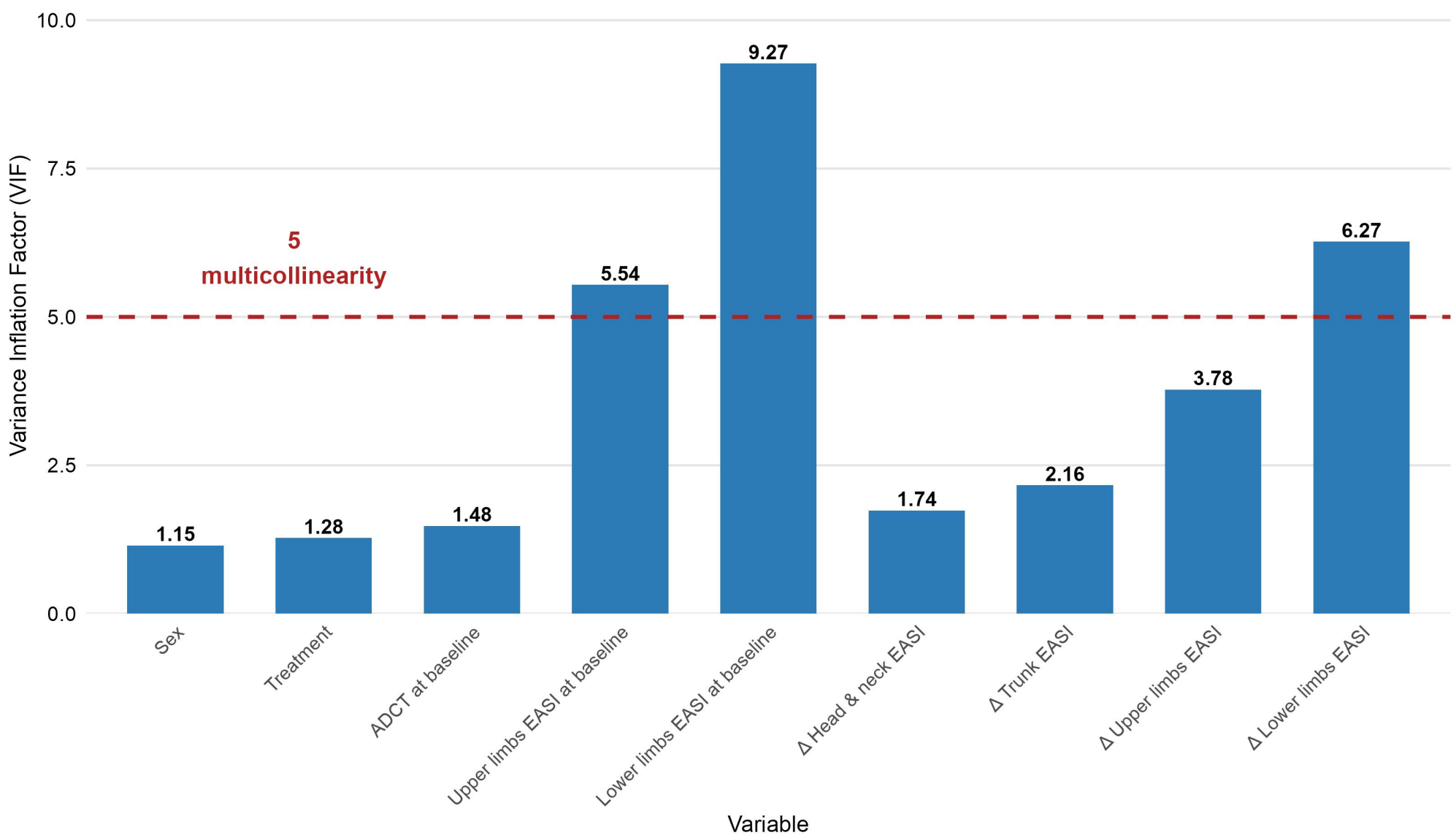

Supplementary Figure 6. VIF for predictors in multivariable logistic regression assessing body region-specific EASI score changes and clinical improvement in AD at Week 24, following LASSO variable selection.

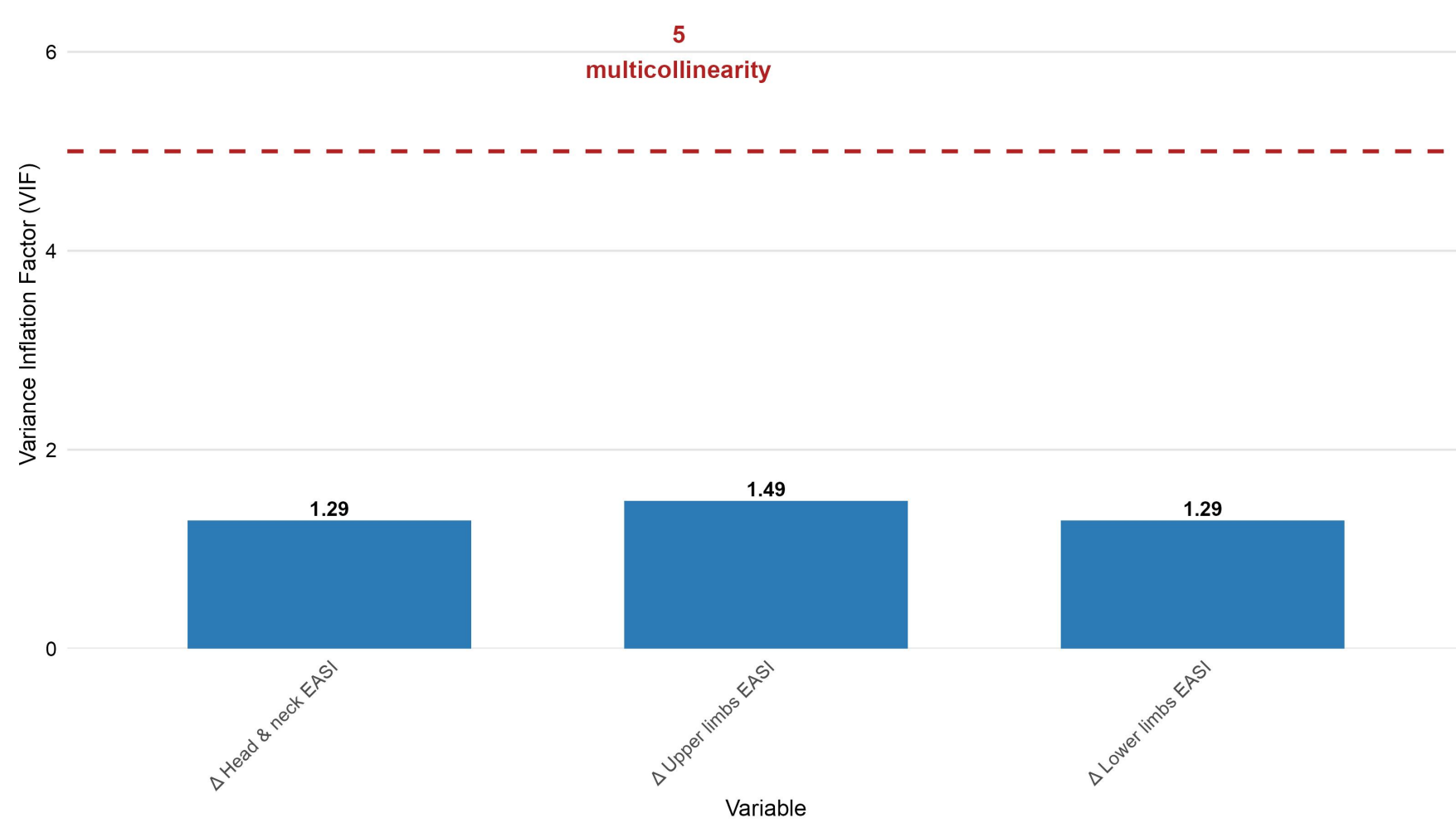

1.2 Supplementary Tables

Supplementary Table 1. Patient demographics and clinical characteristics in our cross–sectional study (*N*=713).

	Total population (N = 713)	Children (N = 175)	Adult (N = 538)

	Total population (N = 713)	Children (N = 175)	Adult (N = 538)
Sex, n/N (%)			
Female	318/713 (44.6%)	97/175 (55.4%)	221/538 (41.1%)
Male	395/713 (55.4%)	78/175 (44.6%)	317/538 (58.9%)
Age	22 (18, 29)	14 (12, 16)	26 (21, 30)
Early onset, n/N (%)	191/434 (44.0%)	55/78 (70.5%)	136/356 (38.2%)
History of atopic diseases, n/N (%)	418/507 (82.4%)	94/111 (84.7%)	324/396 (81.8%)
History of urticaria, n/N (%)	108/522 (20.7%)	9/111 (8.11%)	99/411 (24.1%)
History of allergic rhinitis, n/N (%)	307/521 (58.9%)	85/111 (76.6%)	222/410 (54.1%)
History of asthma, n/N (%)	58/509 (11.4%)	0/111 (0.00%)	58/398 (14.6%)
History of conjunctivitis, n/N (%)	50/521 (9.60%)	4/111 (3.60%)	46/410 (11.2%)
Family history of atopic diseases, n/N (%)	272/521 (52.2%)	71/111 (64.0%)	201/410 (49.0%)
ADCT	7 (4, 12)	7 (3, 11)	7(5, 12)
VAS–Itch	2 (2, 4)	2 (2, 4)	2 (2, 4)
DLQI	7 (3, 11)	6 (3, 9)	7 (3, 11)
POEM	9 (4, 15)	8 (3, 14)	9 (4, 15)
Global EASI	5.60 (2.55, 11.20)	5.10 (2.55, 8.98)	5.88 (2.55, 12.15)

	Total population (N = 713)	Children (N = 175)	Adult (N = 538)
Global EASI severity, n/N (%)			
Mild	413/713 (57.9%)	109/175 (62.3%)	304/538 (56.5%)
Moderate	223/713 (31.3%)	54/175 (30.9%)	169/538 (31.4%)
Severe	77/713 (10.8%)	12/175 (6.86%)	65/538 (12.1%)
Head & neck EASI	5.5 (1, 16)	5.5 (2, 12)	6 (1, 17.75)
Trunk EASI	4 (0, 12)	2 (0, 7.5)	5 (0, 13)
Upper limbs EASI	5 (2, 12)	5.5 (4, 13)	4.5 (2, 12)
Lower limbs EASI	5 (2, 13)	5.5 (3, 12)	4.5 (1.5, 13)

Legends: ADCT: Atopic Dermatitis Control Tool; VAS–Itch: Visual Analog Scale for Itch; DLQI: Dermatology Life Quality Index; POEM: Patient–Oriented Eczema Measure; EASI: Eczema Area and Severity Index.

Supplementary Table 2. Univariate analysis between different variables and inadequately controlled AD.

	Total population (*N*= 713)			
	Children (*N*= 175)		Adult (*N*= 538)	
	OR (95% CI)	*P*	OR (95% CI)	*P*
Age	1.20 (1.02–1.41)	0.024	0.99 (0.97–1.01)	0.489
Sex				
Male	1 [Reference]	0.122	1 [Reference]	<0.001
Female	1.58 (0.96–1.79)		2.45 (1.71–3.52)	
Early onset	0.47 (0.16–1.28)	0.151	1.25 (0.81–1.92)	0.299
History of atopic	0.32 (0.09–0.94)	0.047	0.73 (0.43–1.22)	0.237

	Total population (*N*= 713)			
	Children (*N*= 175)		Adult (*N*= 538)	
	OR (95% CI)	*P*	OR (95% CI)	*P*
diseases				
Family history of atopic diseases	3.81 (1.68–9.13)	0.001	2.08 (1.40–3.09)	<0.001
Head & neck EASI	1.09 (1.04–1.14)	<0.001	1.04 (1.03–1.06)	<0.001
Trunk EASI	1.18 (1.10–1.29)	<0.001	1.06 (1.04–1.08)	<0.001
Upper limbs EASI	1.12 (1.06–1.18)	<0.001	1.08 (1.06–1.11)	<0.001
Lower limbs EASI	1.18 (1.10–1.26)	<0.001	1.12 (1.09–1.16)	<0.001

Note: $P < 0.05$ was considered significant. Abbreviations: EASI: Eczema Area and Severity Index; OR: odds ratio; CI: confidence interval.

Supplementary Table 3. Sensitivity analysis adjusted for global EASI severity.

	Total population (*N*= 713)			
	Children (*N*= 175)		Adult (*N*= 538)	
	OR (95% CI)	*P*	OR (95% CI)	*P*
Age	1.42 (1.05–1.98)[a]	0.026	/	/
Sex				
Male	/	/	1 [Reference]	<0.001
Female	/		4.19 (2.53–7.07)[b]	
History of atopic diseases	0.48 (0.14–1.78)[a]	0.236	/	/
Family history of atopic diseases	10.2 (2.65–54.34)[a]	0.002	1.81 (1.14–2.88)[b]	0.011
Head & neck EASI	1.13 (1.01–1.28)[a]	0.033	1.02 (0.99–1.06)[b]	0.094
Trunk EASI	1.03 (0.85–1.25)[a]	0.758	1.03 (0.99–1.08)[b]	0.101
Upper limbs EASI	0.93 (0.80–1.07)[a]	0.356	1.03 (0.99–1.08)[b]	0.116
Lower limbs EASI	1.12 (0.98–1.28)[a]	0.082	1.13 (1.07–1.20)[b]	<0.001

	Total population (N= 713)			
	Children (N= 175)		Adult (N= 538)	
	OR (95% CI)	*P*	OR (95% CI)	*P*
Global EASI severity				
Mild	1 [Reference]		1 [Reference]	
Moderate	2.63 (0.32–2.44)[a]	0.373	0.83 (0.33–2.02)[b]	0.692
Severe	1.56 (0.93–8.91)[a]	0.989	4.19 (2.53–7.07)[b]	0.040

Note: [a]adjusted for age, history of atopic diseases, family history of atopic diseases, head & neck EASI, trunk EASI, upper limbs EASI, lower limbs EASI and global EASI severity; [b]adjusted for sex, family history of atopic diseases, head & neck EASI, trunk EASI, upper limbs EASI, lower limbs EASI and global EASI severity. $P < 0.05$ was considered significant. Abbreviations: EASI: Eczema Area and Severity Index; OR: odds ratio; CI: confidence interval.

Supplementary Table 4. Baseline ADCT scores stratified by demographic and treatment subgroups in the longitudinal cohort ($N = 96$).

Subgroup	*N*	Mean ± SD	*P*
Age			
Children	26	10.3 ± 4.75	0.084
Adult	70	12.4 ± 6.15	
Sex			
Male	54	11.0 ± 6.21	0.145
Female	42	12.8 ± 5.28	
Early onset			
Yes	35	12.5 ± 5.93	0.385
No	61	11.4 ± 5.83	
History of atopic diseases			
Yes	76	11.8 ± 5.80	0.962
No	20	11.8 ± 6.23	
Family history of atopic diseases			

Subgroup	*N*	Mean ± SD	*P*
Yes	60	11.6 ± 5.52	0.744
No	36	12.1 ± 6.45	
Treatment			
Traditional therapy	34	11.8 ± 6.16	0.998
Targeted systemic therapy	62	11.8 ± 5.73	

Note: $P < 0.05$ was considered significant.

Supplementary Table 5. Baseline characteristics of the longitudinal cohort stratified by treatment group ($N = 96$).

	Traditional therapy ($N = 34$)	Targeted systemic therapy ($N = 62$)	*P*
Sex, n/N (%)			
Female	15/34 (44.1%)	27/62 (43.5%)	1.000
Male	19/34 (55.9%)	35/62 (56.5%)	
Age	24.6 ± 8.87	23.4 ± 9.76	0.523
Early onset, n/N (%)	15/34 (44.1%)	20/62 (32.3%)	0.351
History of atopic diseases, n/N (%)	26/34 (76.5%)	50/62 (80.6%)	0.827
History of urticaria, n/N (%)	6/34 (17.6%)	14/62 (22.6%)	0.759
History of allergic rhinitis, n/N (%)	24/34 (70.6%)	39/62 (62.9%)	0.594
History of asthma, n/N (%)	3/34 (8.82%)	6/62 (9.68%)	1.000
History of conjunctivitis, n/N (%)	2/34 (5.88%)	3/62 (4.84%)	1.000
Family history of atopic diseases, n/N (%)	19/34 (55.9%)	41/62 (66.1%)	0.440
ADCT at baseline	11.8 ± 6.16	11.8 ± 5.73	0.998

	Traditional therapy (N = 34)	Targeted systemic therapy (N = 62)	P
Global EASI at baseline	14.7 ± 13.2	10.1 ± 11.2	0.090
Head & neck EASI at baseline	1.67 ± 1.48	0.99 ± 1.16	0.024
Trunk EASI at baseline	4.93 ± 5.27	2.72 ± 4.00	0.038
Upper limbs EASI at baseline	2.91 ± 2.81	2.03 ± 2.37	0.127
Lower limbs EASI at baseline	5.18 ± 5.73	4.34 ± 5.09	0.479

Note: $P < 0.05$ was considered significant. Abbreviations: EASI: Eczema Area and Severity Index; ADCT: Atopic Dermatitis Control Tool.

Supplementary Table 6. Univariate analysis between different variables and clinical improvement at Week 24.

	Total population (N = 96)	
	OR (95% CI)	P
Age	1.01 (0.97 – 1.06)	0.501
Sex		
Male	1 [Reference]	0.098
Female	0.47 (0.19–1.12)	
Early onset	0.75 (0.30–1.80)	0.536
History of atopic diseases	1.36 (0.48–4.20)	0.570
Family history of atopic diseases	0.95 (0.40–2.28)	0.912
ADCT at baseline	1.06 (0.99–1.14)	0.095
Global EASI at baseline	1.04 (1.01–1.08)	0.029
Head & neck EASI at baseline	1.23 (0.90–1.70)	0.184
Trunk EASI at baseline	1.07 (1.98–1.18)	0.113
Upper limbs EASI at baseline	1.19 (1.01–1.43)	0.040
Lower limbs EASI at baseline	1.10 (1.01–1.20)	0.021
Treatment		0.075

	Total population ($N = 96$)	
	OR (95% CI)	*P*
Traditional therapy	1 [Reference]	
Targeted systemic therapy	2.34 (0.94–6.29)	
Δ Head & neck EASI	1.87 (1.32–2.82)	0.001
Δ Trunk EASI	1.23 (1.09–1.41)	<0.001
Δ Upper limbs EASI	1.59 (1.26–2.13)	<0.001
Δ Lower limbs EASI	1.23 (1.11–1.39)	<0.001

Note: $P < 0.05$ was considered significant. Abbreviations: EASI: Eczema Area and Severity Index; OR: odds ratio; CI: confidence interval.
